# Healthy Lifestyle Interventions for Non-communicable Disease Prevention in Saudi Arabia: A Scoping Review

**DOI:** 10.7759/cureus.99800

**Published:** 2025-12-21

**Authors:** Alla Alhumaid, Abdulaziz Alotaibi, Mashael Tayeb, Yousef Alqhatani, Ibrahim Walbi

**Affiliations:** 1 Obstetrics and Gynecology Department, Imam Abdulrahman Bin Faisal Hospital, Riyadh, SAU; 2 Dental Center, Prince Sultan Military Medical City, Riyadh, SAU

**Keywords:** dietary modification, diet modification, lifestyle intervention, non-communicable diseases (ncds), physical activity

## Abstract

Non-communicable diseases (NCDs), including diabetes, cardiovascular disease (CVD), and obesity, are considered major contributors to mortality and morbidity. Modifiable lifestyle determinants, including unhealthy diets and physical inactivity, play a major role in their progression. Interventions such as lifestyle modification are critical to reduce the onset of NCDs and improve the overall health of the population. This study aimed to synthesize the evidence on lifestyle interventions targeting NCD risk reduction among the population of Saudi Arabia by highlighting the implementing strategies, key outcomes, and recommendations.

A scoping review was conducted following the Preferred Reporting Items for Systematic Reviews and Meta-Analyses extension for Scoping Reviews (PRISMA-ScR) guidelines, given the heterogeneity of the evidence in terms of population, study design, and intervention. Databases searched included PubMed and Google Scholar, yielding 13 studies that met the inclusion criteria. Key outcomes such as improvement in blood pressure, weight, and risk reduction were observed in addition to dietary modification and physical activities that improved health outcomes.

Mindfulness and behavioral strategies support compliance with lifestyle changes. Community initiatives and primary healthcare prevention programs enhance the effectiveness of interventions among various types of populations and those with different levels of education. Culturally tailored and structured programs and customized lifestyle interventions in terms of diet, physical activities, and behavior management or counseling are needed to reduce the incidence of NCDs and their risk factors in Saudi Arabia. Policy intervention to enforce better lifestyle intervention at the national level is needed to mitigate the risks and improve overall health, with prevention and ongoing monitoring considered essential in sustaining long-term health benefits at the population level.

## Introduction and background

Non-communicable diseases (NCDs) such as diabetes, obesity, cardiovascular disease (CVD), and metabolic syndrome represent a major cause of mortality and morbidity across the world and are on the rise in Saudi Arabia, accounting for approximately 73.2% of all mortality and morbidity in the Kingdom [1]. Certain lifestyle risk factors, such as unhealthy dietary habits, smoking, and physical inactivity, exacerbate these conditions. Addressing these modifiable risks through targeted interventions is critical to reduce disease burden and improve population health outcomes [1,2].

Recent studies have emphasized the effectiveness of lifestyle interventions that integrate physical activities, dietary modifications, behavioral strategies, and structured counseling. Furthermore, clinical practice guidelines highlight the importance of personalized approaches that combine exercise counseling, diet, and, when necessary, pharmacological or surgical intervention for the treatment of severe obesity. The Saudi Healthy Plate 2024 (SHP-2024) further provides valuable recommendations in the context of Saudi Arabia [2,3]. However, despite existing guidelines and evidence supporting the effectiveness of lifestyle interventions in Saudi Arabia, several barriers remain, including limited training for healthcare providers, insufficient patient awareness, and low adherence to or compliance with the recommended practices [4]. As a result, public health initiatives play a crucial part in promoting lifestyle modification, particularly among populations at high risk, such as older adults and those with lower levels of education, given that previous studies reported a significant relationship between increased physical activity and optimal weight (p = 0.04) [5-7].

Despite the increasing burden of NCDs in Saudi Arabia, evidence on lifestyle interventions remains fragmented, as existing studies frequently focus on specific diseases, individual behaviors, and/or non-Saudi populations, thereby limiting their relevance to the local context. Therefore, this scoping review aims to summarize, synthesize, and evaluate the current evidence on lifestyle interventions targeting NCD risk reduction in Saudi Arabia by mapping the interventions, key outcomes, and recommendations. Lifestyle interventions are defined as structured or semi-structured programs managing behaviors in terms of dietary modification, physical activity, and weight management, while NCDs refer to chronic conditions including diabetes, cardiovascular diseases, and obesity. While NCDs risk reduction is operationalized as improvements in clinical, behavioral, or metabolic outcomes associated with lower disease risk. By consolidating the evidence, this review provides insights into effective and contextually appropriate strategies to reduce the burden of NCDs and improve population health in Saudi Arabia.

## Review

Methods

Literature Search Strategy

This scoping review was conducted by using major databases and search engines, including PubMed, Web of Science, and Google Scholar, which cover recent studies published from January 2015 to September 2025. The keywords used in the search included “lifestyle interventions,” “physical activity,” “diet modification,” “weight management,” “cardiovascular disease,” “diabetes,” “Saudi Arabia,” and “non-communicable diseases.” In each database and search engine, the Boolean operators (AND, OR) were used to combine keywords and refine the search term (Appendix 1). Additionally, to improve the coverage of local and context-relevant interventions, other sources were included, such as government reports (e.g., SHP-2024) and clinical practice guidelines. Moreover, references from the identified studies were also screened for additional studies that were not captured in the initial search. Title/abstract and full-text screening were conducted by two independent reviewers, and any disagreements were resolved through discussion and consensus.

To assess a wide spectrum of interventions, the scoping review included primary and secondary studies, such as randomized controlled trials, clinical practice guidelines, systematic reviews, and retrospective studies following the Preferred Reporting Items for Systematic Reviews and Meta-Analyses extension for Scoping Reviews (PRISMA-ScR) guidelines. Table 1 shows the elements of the research question, which was developed based on the PICO (Population, Intervention, Comparison, and Outcome) model [8].

**Table 1:** PICO model - item description PICO: Population, Intervention, Comparison, and Outcome; NCD: non-communicable disease

Item	Description
Population	General population of Saudi Arabia
Intervention	Lifestyle interventions such as physical activity, dietary modification, and mindfulness
Comparison	Not appliable
Outcome	Reduction in NCDs and risk factors

Inclusion and Exclusion Criteria

The studies included in this review examined lifestyle interventions implemented in Saudi Arabia, focusing on dietary changes, behavioral counseling, or mindfulness-based programs, and reported effectiveness outcomes such as increased physical activity or reduced risk factors. Studies that focused solely on surgical or pharmacological interventions without a lifestyle component were excluded. Commentaries, editorials, and conference abstracts were also excluded if they lacked sufficient methodological detail or were conducted outside Saudi Arabia.

Data Extraction

A standardized data extraction form was created to systematically capture key information from each study, including year of publication, study design, type of lifestyle intervention, effectiveness outcomes, summary findings, and recommendations. Data extraction was performed independently by two reviewers, with any discrepancies resolved through consensus.

Data Synthesis

A comprehensive narrative synthesis approach was used due to the heterogeneity of the research designs, interventions, and outcomes. This approach highlights the key findings and summary outcomes. The recommendations from each study were integrated to identify and unify common themes and evidence-based guidance applicable to the Saudi population through a word cloud and the development of a theme. This involved developing a preliminary synthesis through structured tabulation, examining patterns and relationships across the studies, and presenting an evidence-based narrative summary in accordance with scoping review methodology and the Popay et al. [9] framework.

Results

Characteristics of Included Studies and Interventions

**Figure 1:**
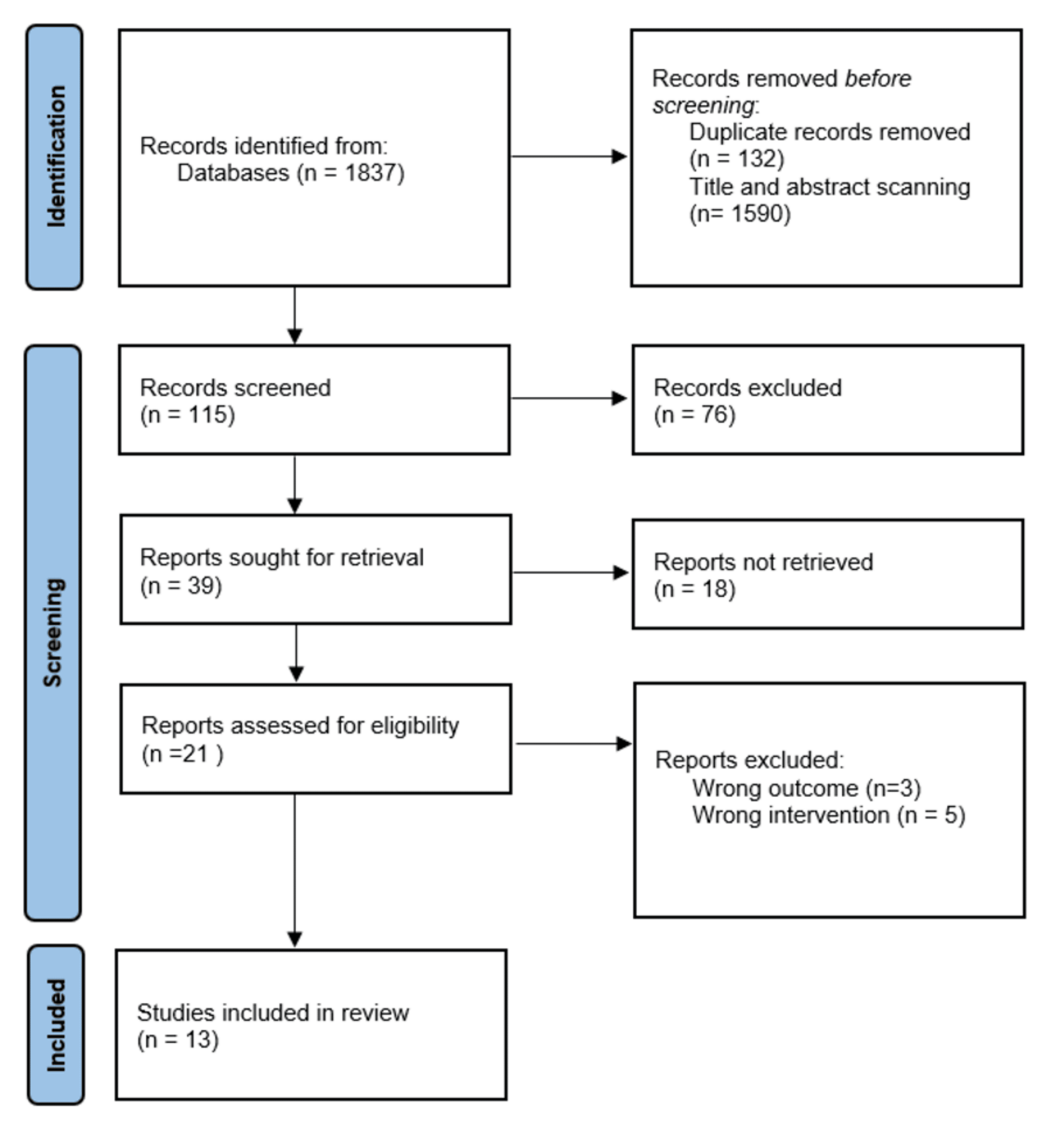
Flow chart depicting the study selection process

This review synthesized findings across 13 studies examining lifestyle interventions for reducing risk factors of NCDs in the context of Saudi Arabia. The studies involved employed diverse research designs such as clinical practice guidelines, systematic review, retrospective design, cross-sectional, and randomized controlled trials (Figure 1).

Among these studies, the most commonly reported lifestyle interventions included physical activities, diet modifications, and structured health education, as well as certain mindfulness practices or cultural programs (Table 2).

**Table 2:** Descriptive characteristics of studies included in the review

Study	Year	Research design	Lifestyle intervention	Effectiveness of intervention	Summary outcome	Recommendation
[10]	2016	Clinical practice guideline	Personalized lifestyle interventions (diet + physical activity)	More effective than usual care; individualized counseling preferred	Strong emphasis on lifestyle interventions with physical activity + diet; conditional use of metformin, orlistat, and bariatric surgery	Recommend lifestyle interventions over usual care, individualized counseling, physical activity with diet, and conditional pharmacological/surgical interventions for obese adults
[11]	2023	Systematic review	Physical activity, healthy diet, mindful meditation	Reduces risk or severity of cardiovascular disease (CVD)	Physical activity, mindful meditation, and diet effectively reduce CVD risk and address physical inactivity and poor diet in the Saudi population	Recommend regular physical activity, mindful meditation, and a balanced diet to reduce CVD risk and address lifestyle-related risk factors
[12]	2025	Retrospective cross-sectional	Exercise (walking) and dietary changes for type 2 diabetes	Positive uptake; improved energy levels and well-being	54.9% exercised regularly, 65.6% made dietary changes; culturally sensitive interventions were effective	Promote culturally sensitive exercise and diet interventions; implement policies supporting lifestyle advice in diabetes programs
[13]	2023	Systematic review	Physical activity, dietary changes, and obesity reduction	Effective for reducing CVD risk	Physical inactivity (69.4%), unhealthy diets (34.4%), obesity, and smoking are major risk factors	Urgently promote lifestyle modifications; increase physical activity, improve diet, reduce obesity; public health campaigns are essential
[14]	2020	Randomized controlled trial	Health education, exercise, and diet counseling	Significant improvements in blood pressure (BP), glucose, Framingham risk score (FRS)	Intervention group reduced systolic BP by 9.2 mmHg, glucose by 45 mg/dL, and FRS by 13.6	Implement personalized lifestyle modification programs with health education, exercise, and diet counseling to reduce CVD risk among women
[15]	2021	Retrospective before-and-after	Tawazon program: diet + physical activity for pre-diabetes	HbA1c, weight, body mass index (BMI), and lipid profiles improved	Phase I & II showed reductions in HbA1c, weight, BMI, and improved lipids	Implement maintenance sessions and online versions to sustain outcomes and expand accessibility
[16]	2022	Systematic review	Physical activity counseling, structured programs	Improved physical activity levels, health parameters	Counseling improved smoking, diet, and exercise habits; barriers include lack of time and training	Train healthcare professionals in physical activity counseling; implement structured programs with educational materials and consultation time
[3]	2025	Clinical practice guideline	Saudi Healthy Plate 2024 (balanced diet)	Nutritionally adequate, culturally appropriate	Meets most macro/micronutrient requirements; gaps in vitamin E and choline	Promote SHP-2024 dietary patterns; future updates should address nutrient gaps (vitamin E, choline)
[17]	2023	Review	Diet + physical activity for obesity	Effective first-line therapy	Low-fat, low-calorie, low-carb, high-protein, low-glycemic index diets were effective; pharmacotherapy/surgery is reserved for later stages	Implement lifestyle management as first-line therapy; encourage exercise and diet; policy interventions to reduce obesity prevalence
[18]	2023	Cohort	Blood pressure lifestyle management (BLSM) program	Mixed outcomes; subgroup improvements	Systolic BP decreased in hyperlipidemia and females; increased in males	Tailor the BLSM program to more participants; increase follow-up frequency for better BP control
[19]	2010	Cross-sectional analysis	Health education via PHC (primary healthcare clinic)	Modest but significant improvements in diet and physical activity	Target vulnerable groups; improved dietary practices and physical activity	Emphasize high-quality health education in PHC; target women, older adults, and those with low education
[20]	2025	Cross-sectional	Physical activity and dietary modifications	Associated with lower metabolic syndrome prevalence	Low activity and poor diet increased risk; interventions needed	Promote physical activity and a healthy diet among adults; implement public health education programs
[21]	2023	Systematic literature review	Dietary modification	Addresses diabetes risk	Increased processed/sugary foods linked to higher diabetes prevalence	Develop interventions targeting dietary habits; promote healthier eating; and public health initiatives to reduce diabetes

Effectiveness of Lifestyle Interventions and Cultural Sensitivity

Clinical practice guidelines emphasize personalized and customized lifestyle interventions, which combine physical activity and diet modification more effectively than usual care. Individualized counseling was also preferred over generic approaches, and surgical options or pharmacological interventions such as metformin or orlistat were suggested for severe obesity among adults [10]. Similarly, the SHP-2024 guideline [3] provided culturally appropriate dietary recommendations that met most macronutrient and micronutrient requirements, though gaps in vitamin E and choline were identified, underscoring the need for targeted updates in future dietary guidelines.

Certain conditions revealed the impact of lifestyle modification on CVD and metabolic health conditions. For instance, one study revealed that physical activity, a balanced diet, and mindfulness mitigated disease severity and reduced CVD risk [11]. Another study reported that unhealthy diets (34.4%), physical inactivity (69.4%), obesity, and smoking were major contributors to CVDs [13]. Moreover, recommendations that were reported to overcome risks and improve health included public health campaigns targeted at lifestyle interventions. Additionally, structured physical activity counseling improved patient adherence to exercise, diet, and smoking habits. Barriers were noted, however, such as training for healthcare providers and limited time [16].

Other research designs, such as cohort studies and randomized controlled trials, provided valuable evidence of intervention effectiveness. For instance, programs such as personalized interventions, including exercise, health education, and diet counseling, resulted in significant improvements in blood glucose (−45 mg/dL), systolic blood pressure (−9.2 mmHg), and Framingham risk score (−13.6) among females at moderate to high CVD risk [14]. Other programs, such as the Tawazon program, produced observable reductions in weight, body-mass index, HbA1c, in addition to lipid profiles in adults with pre-diabetes. Additionally, recommendations were made to maintain online adaptations and sessions to sustain healthy outcomes and enhance accessibility [15].

In terms of cultural sensitivity, one study showed that 54.9% of patients with type 2 diabetes exercised frequently in addition to adopting regular dietary changes (65.6%), which improved overall well-being and energy levels [12]. Another study evaluated a blood pressure lifestyle management program and found positive outcomes overall in terms of improvements in systolic blood pressure among patients with hyperlipidemia, which indicates that a program aiming at lifestyle management can be effective and can vary by demographic and health status [18].

Integration, Public Health, and Sustainability of Lifestyle Interventions

In regard to the primary healthcare role and public health initiatives, health education significantly improved physical activity and dietary practices, in particular among older adults, women, and lower-educated populations. Given the urgency reported in terms of poor dietary habits and low physical activity, which are associated with a higher prevalence of risk factors related to health outcomes, there is a need for targeted public health interventions promoting healthy eating and exercise [19, 20].

Certain recommendations were made to address dietary factors, including the consumption of processed and sugary foods, which contribute to a higher diabetes prevalence. Recommendations include promoting healthier eating habits and controlling diets [21]. Furthermore, combining diet with physical activity serves as a first-line therapy for obesity management, with surgical and pharmacotherapy interventions reserved for severe or treatment-resistant conditions [17].

This evidence collectively indicates that lifestyle interventions that combine and integrate dietary modifications, physical activity, and health education are considered effective in reducing NCDs and the risk factors in Saudi Arabia. Furthermore, structured programs demonstrate robust methods of intervention with the greatest impact. In addition to public health programs, which involve ongoing monitoring and policy support, are essential to sustain long-term population-wide benefits.

Barriers and Core Components of Lifestyle Interventions

Table 3 lists the potential barriers associated with lifestyle modification. These barriers include differences in personal habits, environmental limitations, gaps in the healthcare system, and cultural and traditional factors. For instance, many people struggle with poor motivation due to improper eating habits or physical inactivity. Similarly, healthcare providers frequently lack the necessary expertise or time to provide lifestyle counselling to patients in order to reduce the burden of NCDs and improve care.

**Table 3:** Potential barriers to lifestyle modification Author-generated themes derived from the scoping review findings

Category	Description	Example
Individual factors	Personal limitations that hinder the adoption of healthy behaviors	Lack of motivation; low self-efficacy; poor adherence; limited awareness of disease risks
Behavioral barriers	Habits that conflict with healthy lifestyle choices	Unhealthy dietary habits; physical inactivity; smoking; stress eating
Sociocultural factors	Cultural norms and social expectations influencing behaviors	Social events centered on high-calorie foods; limited culturally tailored dietary guidance; norms discouraging regular exercise (especially among women)
Economic barriers	Financial limitations affecting access to healthy options	Costs of healthy foods; gym memberships; transportation to exercise facilities
Environmental and community factors	Physical environment that limits healthy choices	Limited availability of parks or walkways; lack of safe exercise spaces; limited access to fresh foods
Healthcare system barriers	Challenges within healthcare services that affect lifestyle counseling	Limited counseling time; insufficient provider training; low availability of structured programs; inadequate follow-up systems
Policy-level barriers	Gaps in national or institutional policies that reduce support for lifestyle change	Lack of large-scale public campaigns; inconsistent implementation of guidelines; limited monitoring or incentives
Technological barriers	Issues related to accessing digital or remote health tools	Low digital literacy; limited access to online platforms or apps; poor integration of technology in primary healthcare clinics
Psychological barriers	Mental or emotional obstacles that affect readiness for change	Stress, anxiety, depression, fear of failure, lack of perceived susceptibility to disease
Time and lifestyle constraints	Competing priorities and time limitations	Workload; family responsibilities; irregular schedules preventing routine exercise or meal planning

**Figure 2:**
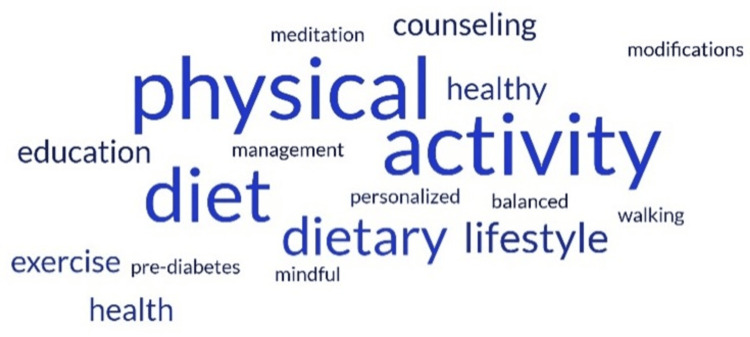
Lifestyle intervention word cloud Author creation via https://www.freewordcloudgenerator.com/generatewordcloud

Figure 2 illustrates the word cloud, which provides the most prominent keywords in terms of lifestyle interventions such as “physical activity”, “diet”, “dietary”, and “lifestyle”, demonstrating the central position of these concepts in this comprehensive review. Furthermore, certain high-frequency keywords reported, such as “healthy”, “balanced”, “personalized”, and “management”, highlight the need for individualized and structured approaches to lifestyle modification. Nonetheless, Additional terms reported, such as “education”, “exercise”, “walking”, “mindful”, “meditation”, and “counseling”, reflect the broader educational components and behavioral acts commonly integrated into lifestyle interventions. While words such as “pre-diabetes”, “health”, and “modifications” suggest a coherent core on risk reduction, NCD prevention, and maintaining the modification of healthy behavior. Overall, the word cloud reflects the research domain dominated by the integration of physical activity and dietary change as foundational pillars of lifestyle management. 

Discussion

Overview of Findings

This scoping review examines lifestyle interventions targeted at the reduction of NCDs and their risk factors in Saudi Arabia. The collective evidence indicates that culturally tailored programs and interventions combining personalized physical activity, structured counseling, and dietary modifications in addition to behavioral strategies are effective in improving health outcomes, including cardiovascular and metabolic outcomes across diverse populations. Interventions involving physical activities were reported to improve energy balance and overall well-being and reduce cardiovascular risk, which corroborates global evidence that physical activities such as exercise are a cornerstone of NCD prevention. Similarly, dietary interventions including culturally adapted guidelines such as the SHP-2024 were reported to be associated with significant improvement in glycemic control, lipid profiles, and weight management, although certain gaps were identified in micronutrient intake [3].

Key Components of Lifestyle Interventions

The terms identified across these studies, such as “physical activity,” “diet,” and “dietary lifestyle,” underscore their importance as cornerstones of lifestyle interventions. Furthermore, their prominence implies a robust consensus that increasing physical activity and modifying nutritional management constitute the major strategies for managing and preventing NCDs, particularly metabolic risk conditions and diabetes. Moreover, the presence of terms such as “personalized,” “healthy,” “balanced,” and “management” points toward a shift to patient-centered approaches, aligning with recent guideline endorsements, which advocate for structured behavioral support and individualized counseling. In addition, keywords such as “counseling,” “education,” “meditation,” “mindful,” and “walking” suggest that lifestyle interventions are increasingly integrating physical, educational, and behavioral activities, which broadens the scope beyond traditional and generic diet and exercise frameworks. These multifactorial interventions, which combine nutrition management, psychological approaches, physical activity, and continual education, have long-term effects.

Effectiveness of Public Health Initiatives and Personalized Interventions

Regarding prevention and management, the inclusion of “pre-diabetes” and “health” shows the significance of these interventions in terms of lifestyle modification, particularly among populations with elevated health risks. Furthermore, the word cloud illustrates and aligns with the literature, reflecting the comprehensive understanding and development of lifestyle intervention concepts as a multidimensional approach in improving the population’s health.

Personalized and customized lifestyle interventions, which incorporated physical activities and diet alongside counseling and behavioral management, contributed to clinically meaningful risk-factor reduction, including cardiovascular factors, BMI, HbA1c, and blood pressure [22]. Similarly, the Tawazon program reinforced the effectiveness of personalized interventions, particularly when supplemented with adaptations and maintained sessions to enhance adherence [3]. Furthermore, mindfulness and behavioral approaches further verified the long-term commitment, reinforcing the benefit of resolving barriers to lifestyle modifications [23].

Public health and primary healthcare service initiatives played a major role in lifestyle management across various populations, in particular among lower-education groups, which highlights the urgent need for targeted interventions for high-risk populations [23]. Various barriers were reported, however, such as a lack of consistent program implementation, inadequate training of the healthcare provider, and low adherence among certain populations, which could reduce the efficiency of interventions [24].

Implications for Policy and Practice

The results provide valuable contributions for policy and practice, given the culturally sensitive nature, in addition to the personalized and structured lifestyle interventions, which promote and prioritize among the community and clinical settings that are supported by health campaigns, health policies, in addition to digital health approaches, which ultimately improve accessibility and sustainability.

*Limitations and Future Research Directions* 

As with any review, the findings are influenced by the availability and quality of the included studies, which varied in design, sample size, and follow-up duration. Additionally, the scope of the search and inclusion criteria may have limited the comprehensiveness of the evidence captured, such as language restriction in English or publication bias. These factors should be considered when interpreting the results, and the findings should be viewed as a broad overview of the current state of knowledge rather than definitive conclusions. Future studies should focus on the analysis of cost-effectiveness and long-term follow-up of interventions that integrate digital and behavioral tools to optimize outcomes and adherence.

## Conclusions

Personalized and contextual lifestyle interventions that integrate physical activity and dietary modification in addition to behavioral and structured counseling can effectively support the reduction of NCDs and their risk factors in Saudi Arabia. Research designs from various intervention types consistently provide evidence for the reduction of risk, such as CVD, weight increase, and high lipid profiles. Moreover, optimal impact can be achieved by combining lifestyle interventions and behavioral strategies, with enhancement in access to primary healthcare and public health initiatives that improve adherence among high-risk groups. To sustain long-term benefits at the population level, however, these interventions must be systematically monitored, and policy implementation at the national level must be improved by leveraging digital health solutions and addressing barriers for healthcare providers and patient adherence in the Kingdom of Saudi Arabia.

## Appendices

**Table 4:** Search strategy and databases used for the scoping review

Date searched	Search string	Database/search engine
2015/01/01-2025/09/30	(("Life Style"[MeSH Terms]OR "lifestyle interventions"[Title/Abstract]) OR ("Motor Activity"[MeSH Terms] OR "physical activity"[Title/Abstract])OR "diet modification"[Title/Abstract]) OR ("Weight Reduction Programs"[MeSH Terms] OR "weight management"[Title/Abstract])) AND (("Cardiovascular Diseases"[MeSH Terms]OR ("Diabetes Mellitus"[MeSH Terms]OR ("Noncommunicable Diseases"[MeSH Terms] AND “Saudi Arabia"[Title/Abstract])	Pubmed
2015/01/01-2025/09/30	TS=("lifestyle interventions" OR "physical activity" OR "diet modification" OR "weight management") AND TS=("cardiovascular disease" OR "diabetes" OR "non-communicable diseases") AND TS=("Saudi Arabia")	Web of Science
2015/01/01-2025/09/30	("lifestyle interventions" OR "physical activity" OR "diet modification" OR "weight management") AND ("cardiovascular disease" OR "diabetes" OR "non-communicable diseases") AND "Saudi Arabia"	Google Scholar
